# Impaired statistical learning of non-adjacent dependencies in adolescents with specific language impairment

**DOI:** 10.3389/fpsyg.2014.00175

**Published:** 2014-03-06

**Authors:** Hsinjen J. Hsu, J. Bruce Tomblin, Morten H. Christiansen

**Affiliations:** ^1^Graduate Institute of Audiology and Speech Therapy, National Kaohsiung Normal UniversityKaohsiung, Taiwan; ^2^Department of Communication Sciences and Disorders, University of IowaIowa City, IA, USA; ^3^Department of Psychology, Cornell UniversityIthaca, NY, USA

**Keywords:** non-adjacent dependencies, statistical learning, specific language impairment

## Abstract

Being able to track dependencies between syntactic elements separated by other constituents is crucial for language acquisition and processing (e.g., in subject-noun/verb agreement). Although long assumed to require language-specific machinery, research on statistical learning has suggested that domain-general mechanisms may support the acquisition of non-adjacent dependencies. In this study, we investigated whether individuals with specific language impairment (SLI)—who have problems with long-distance dependencies in language—also have problems with statistical learning of non-adjacent relations. The results confirmed this hypothesis, indicating that statistical learning may subserve the acquisition and processing of long-distance dependencies in natural language.

## Introduction

In order to correctly interpret a sentence, a language user must often keep track of syntactic dependencies that span across many unrelated words. In English, for example, linguistic material may intervene between auxiliaries and inflectional morphemes (e.g., *is cooking*) or between subject nouns and verbs in number agreement (*the books on the shelf are dusty*). More complex relationships among surface forms are found in long-distance relationships between antecedents and gaps, such as in *wh*-questions (e.g., *Who did you see __?*), anaphoric reference (e.g., *John went to the store where he bought some milk*) and embedded clauses (e.g., *The buildings*_1_ *that the architect*_2_ *built*_2_ *were*_1_ *tall*; where the subscripts indicate dependency relations). Such discontinuous dependencies are considered to be a fundamental and unique property of human language (Tallerman et al., 2009). Indeed, the presence of such non-adjacent relationships in language was a major stumbling block (cf. Chomsky, 1959) for early associationist approaches to syntax (e.g., Skinner, 1957). But does this mean that non-adjacent dependencies cannot be acquired by domain-general means?

Although much statistical learning research has focused on the detecting dependencies between adjacent linguistic elements (Gómez and Gerken, 2000; Saffran, 2003, for reviews), relatively little research has focused on the learning of non-adjacent syntactic relationships. A key exception is recent work indicating that statistical learning of non-adjacent dependencies improves as the variability of elements that occur between two dependent items increases (Gómez, 2002; Onnis et al., 2003, 2004). When the set of items participating in the dependency is small relative to the set of intervening elements, the non-adjacent dependencies stand out as invariant structure against the changing background of more varied material. In addition, statistical learning of non-adjacencies has been demonstrated both for non-linguistic sounds (e.g., Gebhart et al., 2009) and visual stimuli (e.g., Fiser and Aslin, 2001, 2002; Onnis et al., 2003; Conway and Christiansen, 2006; Pacton and Perruchet, 2008), suggesting that such learning was supported by domain-general mechanisms. However, an important theoretical caveat remains: it is unclear whether the mechanisms involved in such *variability learning* are also used for non-adjacencies in language. Indeed, the potential relevance of statistical learning for understanding syntactic aspects of language has been the subject of much debate (e.g., Musso et al., 2003; Friederici, 2004—but see Marcus et al., 2003; de Vries et al., 2008). In this paper, we test whether the same mechanism underlying variability learning also subserves natural language learning. This hypothesis will be tested by investigating whether individuals with SLI, who have well-attested difficulties with long-distance dependencies (e.g., Clahsen et al., 1997; Wexler, 2000; van der Lely and Battell, 2003), also have problems using variability to learn non-adjacent dependencies.

Children's sensitivity to non-adjacent dependencies in language emerges gradually, with those apparent in the surface structure of sentences acquired earlier than more abstract non-adjacencies. For example, 18-month-olds are sensitive to violations of non-adjacent dependencies between *is* and *-ing* in comprehension (Santelmann and Jusczyk, 1998), and the use of the present progressive morpheme *-ing* also shows up early in production (though initially without the appropriate dependency relation to the auxiliary *is*; Brown, 1973). Children's ability to deal with more abstract non-adjacencies comes later. Even after they have otherwise mastered subject-noun/verb agreement around 2–2.5 years of age, they still produce incorrect *wh*-questions with agreement violations (such as, ^*^What color is these?; Radford, 1990). Moreover, children also have problems responding correctly to *wh*-questions involving a direct object *wh*-word and a non-copular verb (such as, *What did mummy say?* to which a 21-month-old responded *Mummy*; Radford, 1990). From age 3 years and onward, children start to produce sentences of increasing length and syntactic complexity, such as coordinating conjunctions and center-embedded sentences in which the main clause is interrupted by a relative clause. Production and comprehension errors of embedded relative clauses are still frequent in children aged between 3 and 6 years (Gaer, 1969; Cook, 1973).

The order of acquisition of non-adjacencies in natural language suggest that dependencies governing subject-noun/verb agreement and auxiliary/inflectional morpheme relations—which primarily involve surface-level cues between functional elements—are acquired earlier than non-adjacent dependencies involving more abstract constituent relationships, such as those found in *wh-*questions and embedded relative clauses. Thus, work on the statistical learning of non-adjacencies between words^1^ has focused on non-adjacent dependencies discernable in surface-level information. Gómez (2002) and Onnis et al. (2003, 2004) exposed adults to artificial languages in which sentences took the form of *aXd*, *bXe*, and *cXf* (e.g., *pel*-*wadim*-*rud*). Drawing on the observation that certain elements in natural language belong to relatively small sets (function morphemes like *a*, *was*, *-s*, and *-ing*), whereas others belong to very large sets (nouns and verbs), and the fact that learners must often track key dependencies between functional elements, the experimenters manipulated the size of the set from which the intervening *X*-elements were drawn. The hypothesis was that increasing the variability of the middle element would cause learners to steer away from adjacent dependencies (e.g., *aX* and *Xd* in the string *aXd*) and instead focus on the non-adjacent *a–d* relationship. Variability was manipulated by drawing *X* from a set containing 2, 6, 12, or 24 elements. Participants were then tested on their abilities to distinguish sentences from the language (e.g., *aXd*) from foils (e.g., *aXe*). Counterintuitively, participants acquired the non-adjacent dependencies only when the variability of the middle items was at its highest (in set-size 24). Note that associations between adjacent elements cannot explain these results because first-order conditional probabilities, e.g., P(*X*|*a*), decrease as the set size of *X* increases. Hence, participants only learned non-adjacent dependencies when adjacent dependencies were least predictable. Additional experiments demonstrated that infants as young as 15 and 18 months of age (Gómez, 2002; Gómez and Maye, 2005) are able to use variability learning to discover non-adjacent dependencies, suggesting that this type of learning is present from at least the middle of the second year of life.

The positive effect of high variability has been replicated in several subsequent studies. Misyak and Christiansen (2012) obtained significant learning using a set-size of 24 and found that individual differences in such learning correlated with offline language comprehension of sentences involving embedded relative clauses. By incorporating the set-size 24 stimuli within a serial-reaction time (SRT) task, Misyak et al. (2010a,b) also replicated the effect of high variability. They further found that performance on this non-adjacency learning task predicted online processing of embedded relative clauses in natural language. More generally, it seems, though, that for non-adjacent dependency relations to be learnable, some facilitatory factor is necessary, such as high variability (as investigated here), phonological or visual cues (e.g., de Vries et al., 2012; van den Bos et al., 2012), scaffolded learning (Lai and Poletiek, 2011), or prolonged exposure (Udden et al., 2012). Some combination of these facilitatory factors are likely to be available in language development, suggesting a possible role for statistical learning in guiding the first steps of acquisition of not only simple but also the more complex, non-adjacent syntactic structures.

Children with SLI provide an ideal population to test the hypothesis that statistical learning and language are supported by the same underlying mechanisms. These children present a slow development of spoken language that in most cases results in long-term restrictions in listening and speaking skills in the absence of hearing loss, or other neurodevelopmental disorders, including autism and mental retardation (Tomblin et al., 1996). Extensive research has shown that children with SLI have considerable difficulties with the grammatical morphology of English (e.g., Johnston and Schery, 1976; Gopnik and Crago, 1991; McGregor and Leonard, 1994; Hadley and Rice, 1996; Cleave and Rice, 1997; Bedore and Leonard, 1998) and other languages (e.g., Clahsen, 1989; Leonard, 2000)—in particular, with grammatical relationships extending across non-adjacent lexical elements within and between clauses. These difficulties with long-distance syntactic dependencies have been addressed within generative grammar perspectives by Wexler's (2000) Unique Checking Constraint account of SLI, van der Lely and Battell's (2003) representational deficits for long-distance relationships theory, and Clahsen et al.'s (1997) agreement-deficit hypothesis. These accounts have explained the difficulties children with SLI have with long-distance dependencies in terms of domain-specific grammatical impairments. In contrast, we hypothesize that impairments to statistical learning mechanisms supporting variability learning underlie these observed problems with non-adjacent dependencies in language.

Preliminary support for this hypothesis comes from studies investigating statistical learning of adjacent dependencies. Evans et al. (2009) reported that children with SLI were unable to use transitional probabilities between adjacent syllables to identify word boundaries. Additional support comes from two studies involving a heterogeneous population of college-aged adults with a history of language impairment, dyslexia, and/or learning disabilities (LI/D/LD), for which they have received therapy and/or other service. Individuals with LI/D/LD were found to have problems not only in using adjacency information to learn word patterns generated by a finite state grammar (Plante et al., 2002) but also with variability learning of non-adjacent dependencies (Grunow et al., 2006).

Given that statistical learning involves implicit learning of probabilistic patterns, research on procedural learning in SLI also casts light on our hypothesis. Several SRT studies observed poorer learning of sequences of visual patterns in children with SLI (Tomblin et al., 2007; Lum et al., 2010, 2011; Hedenius et al., 2011). Moreover, Kemeny and Lukacs (2009) reported depressed performance by children with SLI on a Weather Prediction Task that involves learning probabilistic classification. Thus, whereas previous studies point to a possible link between statistical learning and language ability, we provide a direct test of the account by determining whether individuals with SLI—who have well-attested problems with syntactic non-adjacencies—also have problems using variability learning to discover such dependencies via statistical learning. To this end, we adopted the non-adjacent dependency learning task developed by Gómez (2002) and compared performance of a group of adolescents with SLI to adolescents with normal language (NL) ability. We predicted that high variability of the intervening elements would facilitate the NL learners' learning of non-adjacent dependencies, but would not aid the SLI learners.

A second goal of the current study was to gain further understanding of the learning processes involved in learning non-adjacent dependencies, particularly in individuals with SLI. Previous studies have indicated that high variability may not facilitate learning of non-adjacent dependencies in individuals with language impairment. This suggests that learners with language learning difficulty might exploit a different learning strategy that is sensitive to the number of target pairs to learn. It is possible that the participants with language learning difficulty learned the sentence strings exemplar by exemplar without paying attention to the structural regularities embedded in the stimuli. In the current study, we investigated this hypothesis by examining the accuracy of each target non-adjacent pairs separately.

## Methods

### Participants

One hundred twenty adolescents aged 13–15 years were recruited from a large sample of children who have been participating in a longitudinal investigation of SLI (see Tomblin et al., 1997, for details of sampling and assessment). Sixty of these adolescents had NL skills and 60 were age- and non-verbal IQ-matched adolescents with specific language impairment (SLI)^2^. The participants from each language group (NL, SLI) were randomly assigned to one of three variability conditions: low (*X* = 2), mid (*X* = 12), and high (*X* = 24) variability. Two-Way ANOVAs, with groups and variability conditions as the between-subject factors, were conducted to inspect group differences in non-verbal IQ and language abilities between groups across different conditions. Group summary statistics are provided in Table 1. Each of the SLI groups had comparable non-verbal cognition to the paired NL groups in terms of Performance IQ on WISC-III (Wechsler, 1991; *F*_(1, 114)_ = 0.12, *p* = 0.74), but showed significantly poorer language abilities than the paired NL groups in terms of language composite standard scores [*F*_(1, 114)_ = 137.6, *p* < 0.0001] compiled from CELF-III (Semel et al., 1995), PPVT-R (Dunn and Dunn, 1981), CREVT (Wallace and Hammill, 1997), and the listening comprehension adaptation of the QRI-II (Leslie and Caldwell, 1995). Differences in non-verbal cognition and language composite scores between the three SLI subgroups or between the three NL subgroups were not significant. Scores from the Competing Language Processing Task-Word Repetition subtest (CLPT-Word Repetition, Gaulin and Campbell, 1994) did not serve as a selection criterion but were used to test potential effects of working memory on non-adjacency learning. Informed consent was obtained from each of the participants before they took part in the current study. This research was approved by the Institutional Review Board of the University of Iowa.

**Table 1:** **Group summary statistics for the adolescents with specific language impairments (SLI) and with normal language (NL) in the low (*X* = 2), mid (*X* = 12), and high (*X* = 24) variability conditions**.

		**Age**	**CLPT**^a^****	**WISC-III**^b^****	**Language composite score**
*X* = 2	NL	13;9 (0.6)	72.8 (13.6)	95.1 (11.0)	97.6 (10.1)
	SLI	14;2 (0.6)	56.6 (11.2)	94.1 (12.8)	76.7 (6.8)
*X* = 12	NL	14;2 (0.5)	73.4 (12.3)	94.3 (13.3)	96.8 (12.6)
	SLI	14;1 (0.6)	62.2 (11.3)	93.40 (12.6)	77.01 (7.5)
*X* = 24	NL	14;3 (0.6)	74.8 (8.4)	95.9 (12.2)	96.2 (11.0)
	SLI	13;8 (0.4)	58.6 (13.8)	95.5 (10.4)	76.9 (6.2)
Total	NL	14;1(0.6)	73.7 (11.4)	95.1(12.0)	96.9(11.1)
	SLI	14;0 (0.6)	59.1 (12.2)	94.3(11.8)	76.9 (6.8)

CLPT, WISC-III, language composite scores: standard scores with a mean of 100, standard deviation of 15.

a Competing Language Processing Task-Word Repetition subtest.

b Wechsler Intelligence Scale for Children (3rd edition)-Performance IQ.

Standard deviations are reported in parentheses.

### Materials

Following Gómez (2002), the stimuli consisted of three dependency pairs: *aXd, bXe*, and *cXf*. To investigate the role of variability in non-adjacency learning, we varied the size of the set from which the middle element (*X*) was drawn: low (*X* = 2), mid (*X* = 12), and high (*X* = 24) variability. The beginning (*a, b, c*) and ending (*d, e, f*) stimulus tokens were instantiated by the non-words *pel*, *dak*, *vot*, *rud*, *jic*, and *tood.* The non-words used to instantiate the 24 intervening *X*-tokens in the high-variability conditions were *wadim*, *kicey*, *puser*, *fengle*, *coomo*, *loga*, *gople*, *taspu*, *hiftam*, *deecha*, *vamey*, *skiger*, *benez*, *gensim*, *feenam*, *laeljeen*, *chila*, *roosa*, *plizet*, *balip*, *malsig*, *suleb*, *nilbo* and *wiffle*. The *X*-tokens for the low- and mid-variability conditions consisted of the first 2 and 12 non-words, respectively, from this set. Each non-word was recorded separately by a female native speaker of English to ensure that lexical stress was similar for all monosyllables and all disyllables. The assignment of particular tokens (e.g., *pel*) to particular stimulus variables (e.g., the *b* in *bXe*) for each participant was randomized to avoid learning biases due to specific sound properties of the non-words (Onnis et al., 2005). There was a 250-ms pause between each word in a string, and a 750-ms pause between strings.

Frequency of exposure to the dependency pairs (i.e., *aXd, bXe*, and *cXf*) was held constant across the three variability conditions, allowing for comparisons of learning in the three variability conditions. The training stimuli consisted of 144 presentations of each dependency pair, randomly interleaved, for a total of 432 training strings. The test material included 6 instances of the original training strings (two each of *aXd, bXe*, and *cXf*) and 6 foils produced by disrupting the non-adjacency relationship (two each of ^*^aXe, ^*^bXf, and ^*^cXd).

### Procedure

Twenty participants from each language group (NL, SLI) were randomly assigned to one of the three variability conditions. They were instructed to listen to sequences of non-sense syllables, the knowledge of which they would later be tested. The participants were not informed about any rules or patterns embedded in the materials^3^. After training participants were informed that the syllable sequences they heard were generated according to rules specifying word order and asked to provide grammaticality judgments for the test items by pressing a Y (Yes for grammatical strings) or a N (No for ungrammatical strings) key on the keyboard.

## Results

### Overall performance

**Figure 1:**
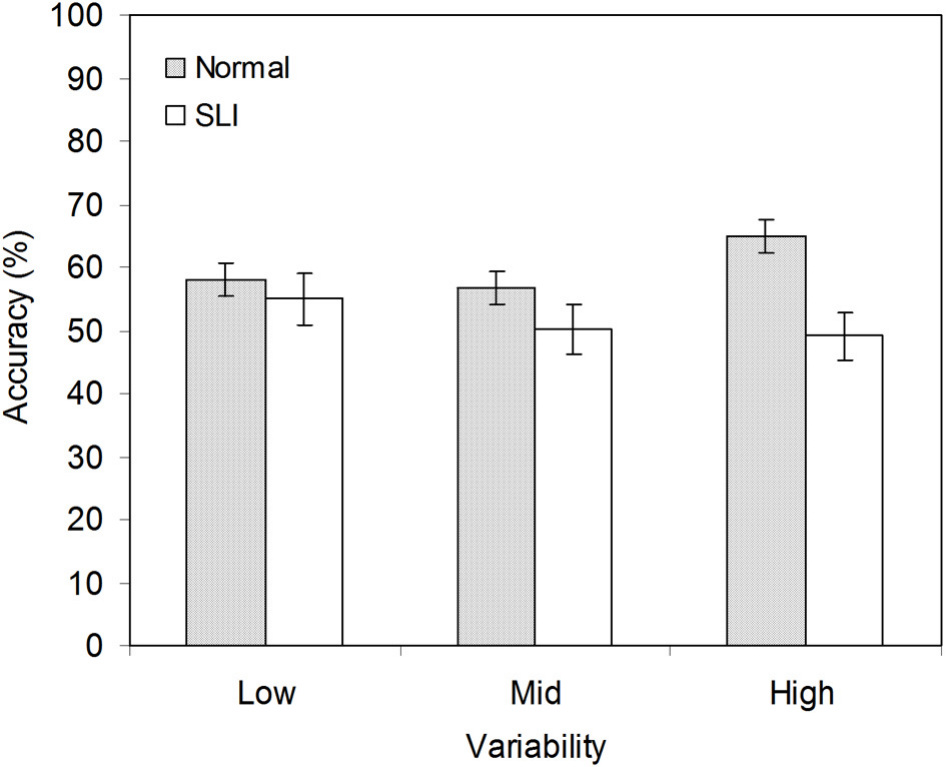
**Mean accuracy for the NL and the SLI group in the low, mid, and high variability conditions. Error bars represent s.e.m**.

The overall mean accuracy scores of the SLI and the NL groups in each of the three variability conditions is shown in Figure 1. There were a total of 12 test items, of which 6 contained grammatical strings and 6 ungrammatical strings.

A list of each participant's performance in terms of hit (i.e., the proportion of endorsements for grammatical items) and false-alarm (i.e., the proportion of endorsements for ungrammatical items) rates is provided in the Table A1^4^. Table 2 presents group mean accuracy of hits and false alarms for the SLI and the NL groups. First, we inspected response bias (β) across groups and variability conditions. We found that group difference in β were not significant in any one of the three variability conditions [*t*_(38)_ = −0.70, *p* = 0.46 in *X* = 2; *t*_(38)_ = 0.80, *p* = 0.43 in *X* = 12, *t*_(38)_ = 1. 43, *p* = 0.16 in *X* = 24]. Given that the two groups did not show different response biases, the participants' performance was evaluated statistically using a mixed design ANOVA with language group (NL vs. SLI) and variability condition (*X* = 2, *X* = 12, *X* = 24) as between-subjects variables and grammaticality (grammatical vs. ungrammatical strings) as a within-subjects variable. There was a significant main effect of grammaticality, *F*_(1, 114)_ = 11.43, *p* = 0.001, partial η^2^ = 0.09, and Grammaticality × Language Group interaction, *F*_(1, 114)_ = 6.34, *p* = 0.01, partial η ^2^ = 0.05. There were no other main effects or interactions. *Post-hoc* comparisons indicated that overall the NL learners accepted grammatical strings more frequently than they accepted ungrammatical items [*t*_(59)_ = 3.97, *p* < 0.001, *d* = 0.80]. However, this pattern of performance was not observed in learners with SLI.

**Table 2:** **Participants' responses in terms of hit and false-alarm rates for the NL and the SLI groups**.

**Set size**	**NL**			**SLI**		
	**Hit**	**False positive**	**Difference**	**Hit**	**False positive**	**Difference**
2	0.75 (0.05)	0.59 (0.05)	0.16	0.68 (0.04)	0.58 (0.03)	0.10
12	0.70 (0.6)	0.57 (0.07)	0.13	0.61 (0.06)	0.61 (0.06)	0.00
24	0.79 (0.06)	0.49 (0.06)	0.30	0.60 (0.08)	0.61 (0.07)	−0.01

The numbers in parentheses represent standard errors of the mean.

We predicted that high variability of the intervening elements would facilitate the NL learners' learning of non-adjacent dependencies, but would not aid the SLI learners. To test this prediction, we conducted a series of planned comparisons to examine the rates of acceptance of grammatical strings against ungrammatical strings for the two groups in each of the three variability conditions. There was a significant grammaticality effect with a large effect size for the NL learners exposed to high variability [*t*_(19)_ = 3.01, *p* = 0.007, *d* = 1.06]. In addition, a significant grammaticality effect with a moderate effect size was observed for the NL learners exposed to low variability [*t*_(19)_ = 2.14, *p* = 0.046, *d* = 0.65]. The decrease in effect size suggests that high variability best facilitates learning of non-adjacent dependencies. In contrast, performance by the learners with SLI did not reach significance in any variability condition. Together, the results suggest that high variability facilitates non-adjacent dependencies learning for NL learners, but not learners with SLI.

**Figure 2:**
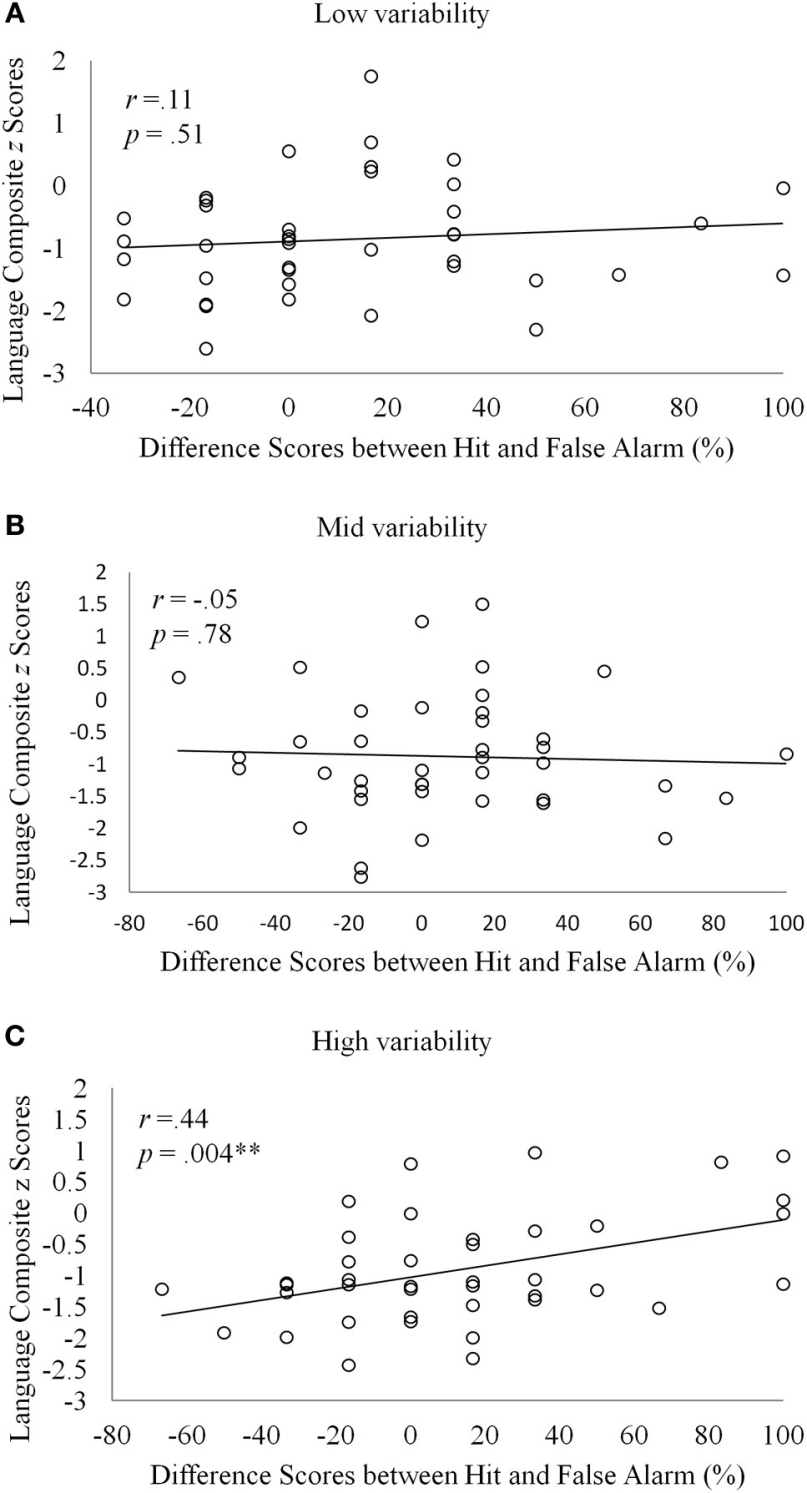
**Scatter plots of language composite *z* scores and the difference scores between hit and false alarm for the (A) low, (B) mid, and (C) high variability conditions**.

Might there be a correspondence between individual differences in learning non-adjacent dependencies and individual variations in language skills across the two groups? Specifically, if high variability is critical for detecting and learning dependent relationships between remote items, we might expect to see an association between the participants' language skills and their performance in the high variability conditions. Simple correlations (Pearson's *r*) were calculated between the participants' language composite scores and the difference scores between correct acceptance and false positives in the non-adjacent dependency learning task. As shown in Figure 2, a significant, albeit modest, correlation was found for high variability (*r* = 0.44, *p* = 0.004), indicating a positive relationship between the ability to learn non-adjacent dependencies under high variability and language attainment. Non-significant correlations were obtained for the other two variability conditions. Moreover, the difference scores in the high variability condition were not significantly correlated with individual differences in working memory measured with CLPT.

### Item-specific learning in SLI

Although the SLI participants as a group did not show evidence of learning in any variability condition, it remains possible, though, that some non-adjacent pairs were learned by SLI learners, but that the aggregate across items was not great enough to show a significant learning effect. We therefore calculated the number of non-adjacent word pairs (max = 3) that each participant learned. A given non-adjacency pair is considered “learned” if a learner was able to correctly accept all grammatical and reject all ungrammatical strings involving this pair (i.e., hit rate = 100% and false positive = 0%). This scoring method allows us to examine item specific learning that might be obscured by the aggregate score. Furthermore, such item specific learning may benefit more from low variability where fewer items need to be learned.

**Figure 3:**
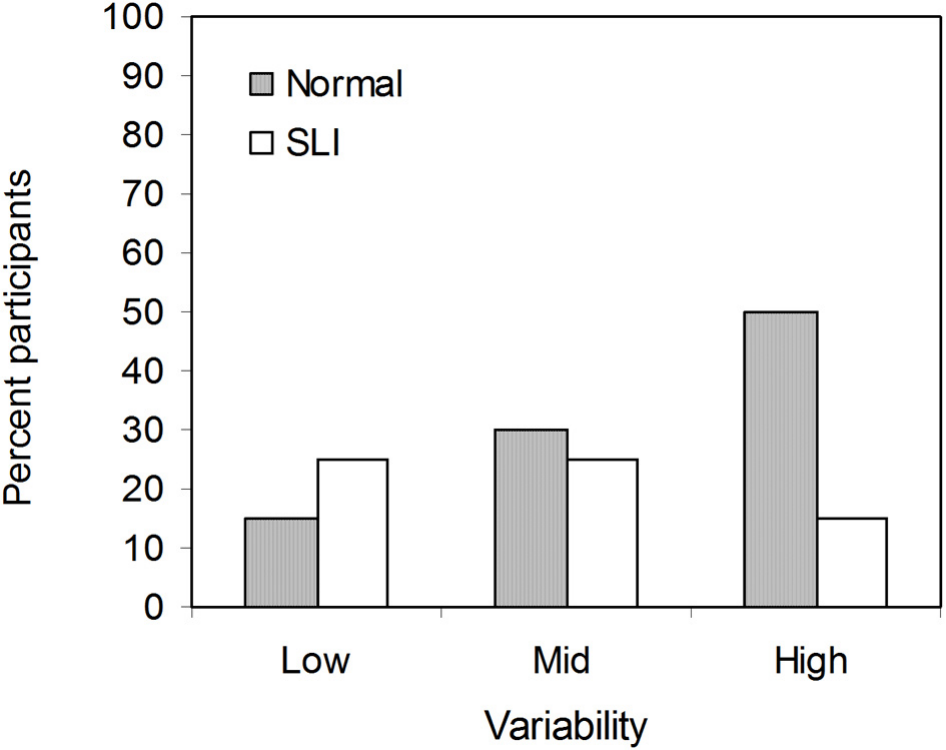
**Percent participants in the NL and the SLI group learned at least one non-adjacent pairs in the low, mid, and high variability conditions**.

**Figure 4:**
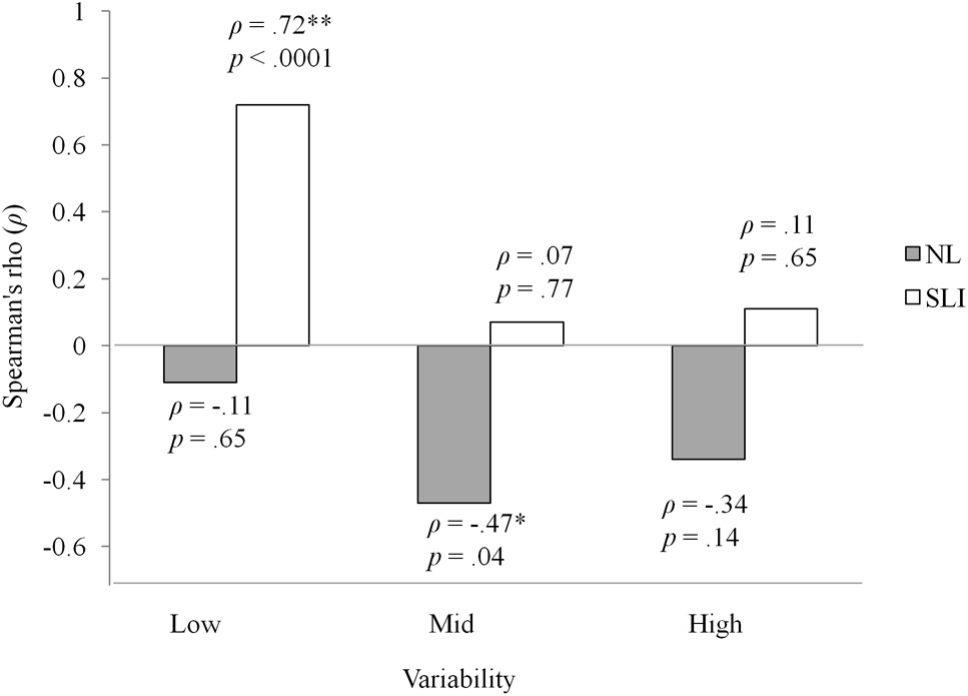
**Correlations between number of pairs learned and language ability for the pal1icipants with SLI in the low, mid, and high variability conditions**.

Figure 3 shows the percentage of participants who learned at least one non-adjacent pair in each group and variability condition. Interestingly, the proportion of the SLI participants who learned at least one non-adjacent pair under low variability was slightly higher than that under high variability, while the opposite was true for the NL group. The finding that more participants with SLI benefitted from low than high variability in learning non-adjacency pairs suggests item-specific learning: in the low variability condition there were 6 different strings (*pel-wadim-rud, pel-kicey-rud, dak-wadim-jic, dak-kicey-jic, vot-wadim-tood, vot-kicey-tood*), each of which occurred for 72 times (i.e., high token frequency), whereas in the high variability condition there were a total of 72 different strings, with each string occurring only 6 times (i.e., low token frequency). We further explored this suggestion by examining correlations between language and performance in learning non-adjacent dependencies. A list of each participant's language composite score and number of non-adjacent item mastered is provided in the Table A2. Because there were participants who did not reach 100% accuracy on any of the three non-adjacent pairs, Spearman's rank correlation coefficient was used to minimize the effect of extreme scores. Strikingly, as illustrated in Figure 4, there was a significant correlation for the SLI group under low variability (ρ = 0.72, *p* < 0.0001). That is, within the SLI group, those who had better language ability learned more pairs under low variability than those who had poorer language ability. No correlations were found for mid and high variability. For the NL group, the correlation coefficients in all three conditions were negative, only just reaching significance in the mid variability condition (ρ = −0.47, *p* = 0.04). Thus, for NL participants, better language ability was not associated with mastering non-adjacency pairs—indeed, there was a trend in the opposite direction.

## Discussion

The current study investigated variability learning of non-adjacent dependencies in adolescents with and without SLI. For the adolescents with NL ability, both those exposed to the high and low variability conditions showed an effect of learning, but the relative effect sizes suggest that high variability best facilitates learning of non-adjacent dependencies. It is possible that for the NL learners, repeated exposure to a few unique exemplars in the low variability condition could also assist learning. Importantly, though, the correlation analyses showed that only performance under high variability was associated with an individual's language skills. For the learners with SLI, on the other hand, performance in the high variability condition did not reach significance. Thus, although both infants and typically-developing adults are able to use variability learning to detect non-adjacent dependencies in speech input (Gómez, 2002; Onnis et al., 2003; Grunow et al., 2006), the SLI group was unable to do so.

The same-mechanism hypothesis predicts an association between the participants' language skills and performance in the non-adjacent task. In the current study, we found a significant, albeit modest, correlation between the two variables in the high variability condition. That the association was only moderate might reflect the fact that the participants' language skills were evaluated using composite scores that pooled across several standardized language tests, rather than using tests specifically designed for evaluating syntactic performance on non-adjacent structures in English. Future studies should use tests that more directly examine individuals' proficiency in non-adjacent structures in their native language (e.g., as in Misyak et al., 2010a,b).

Why did the SLI participants fail to show learning under conditions for which their NL peers did learn? Analyzing the dependency-pair mastery scores, we found different group profiles across the three variability conditions. For the NL group, high variability of the intervening elements led to the best mastery scores. In contrast, more non-adjacent pairs were learned by SLI adolescents under low variability than high variability, suggesting that perhaps different types of learning, involving different kinds of statistics, were adopted by the two groups in learning non-adjacent word pairs.

One possible interpretation of the observed difference in learning pattern is that the adolescents with SLI might have attempted to learn the materials by rote memorization. Given that the low variability condition only involves 6 individual strings, each presented 72 times, whereas the high variability condition incorporated 72 separate strings, each presented only 6 times, such an approach would seem reasonable. However, given that typically-developing adults are able to generalize to novel strings—even when exposed to a zero variability condition with only 3 unique strings—statistical learning of non-adjacencies is unlikely to involve memorization under normal circumstances (Onnis et al., 2004). In contrast, the SLI group may have sought to memorize the strings, consistent with evidence that children with SLI rely substantially on memorized surface properties in spontaneous speech (e.g., Jones and Conti-Ramsden, 1997; Riches et al., 2006). Thus, the correlation we found between number of adjacency pairs learned and language ability may suggest that memorization of input chunks as unanalyzed wholes may provide some advantages as a compensatory strategy for language learning and processing, even though it may impede statistical learning of more complex aspects of language, including non-adjacent dependencies.

Tracking remote dependencies is a crucial for language acquisition. In this study, we have shown that the well-documented problems that individuals with SLI have with long-distance syntactic dependencies may be associated with their inability to take advantage of variability in statistical learning. Given that statistical learning of non-adjacencies has been demonstrated both for non-linguistic sounds (e.g., Gebhart et al., 2009) and visual stimuli (Onnis et al., 2003; Pacton and Perruchet, 2008), the SLI participants' problems with the non-adjacency learning task may reflect an impairment of domain-general mechanisms hypothesized to play an important role in the acquisition and processing of discontinuous dependencies in natural language. In typically-developing individuals, these mechanisms allow learners to use additional cues to acquire both probabilistic non-adjacencies (van den Bos et al., 2012) as well as multiple overlapping non-adjacent dependencies (de Vries et al., 2012). More generally, this study contributes to our emerging understanding of the interrelationship between statistical learning and language in typically-developing populations (e.g., Misyak et al., 2010a,b; Misyak and Christiansen, 2012), while underscoring the need for additional research on the possible role of statistical learning deficits in SLI.

### Conflict of interest statement

The authors declare that the research was conducted in the absence of any commercial or financial relationships that could be construed as a potential conflict of interest.

## Footnotes

^1^The learning of non-adjacency relationships between syllables within words have yielded mixed results (e.g., Peña et al., 2002; Onnis et al., 2005), though evidence of sensitivity to nonadjacent dependencies between phonological segments has been found (Newport and Aslin, 2004). Moreover, Onnis et al. (2003) have subsequently demonstrated that it is possible to learn dependencies between non-adjacent syllables within words when the syllabic material intervening the dependent syllables is highly variable.

^2^Participants in both groups were not required to have performance IQ levels above 85 in this study. Although this restriction has been common in SLI studies, it has recently come under scrutiny (Tager-Flusberg and Cooper, 1999). Thus, as shown in Table 1, 25% of the children with SLI had performance IQs below 85. Crucially, the two groups differed only on language skills, whereas the performance IQ was the same across both groups.

^3^We used the same instructions as in Gómez (2002): “Your task is to listen to sequences of non-sense syllables. We will test you later so pay close attention. This phase of the study takes about 20 min, divided into 3 parts. You can take a break after each part. Please let the experimenter know if you have any questions.”

^4^Using the criteria of hit rate at or below 0.33 and a false-alarm rate at or above 0.66, we found that in the high variability condition there were five participants with SLI but only one participant with NL who showed a low rate of hits but high rates of false alarms. Crucially, such group difference was not found in the other two variability conditions (only 1 participant from each group in the *X* = 2 condition, and 2 from the NL and 3 from the SLI groups in the *X* = 12 condition). Therefore, the observed group difference in the *X* = 24 condition is unlikely to derive from overall confusion about performing the task, but instead might reflect general difficulty in learning nonadjacent pairs in the learners with SLI.

## Appendix

**Table A1:** **Hit and false-alarm rate for each participant in the three conditions**.

	*****X*** = **2****		*****X*** = **12****		*****X*** = **24****	
	**Hit**	**False positive**	**Hit**	**False positive**	**Hit**	**False positive**
NL	0.83	0.67	0.83	0.67	0.83	0.83
	1	0	0.83	0.67	1	1
	0.67	0.50	0.67	0.67	1	0
	1	0.17	0.50	0.67	0.67	0.33
	0.33	0.67	0.67	0.67	1	0.50
	0.83	1	0.50	0.67	0.33	0.67
	0.67	0.50	0.67	0.50	0.83	0.50
	0.67	0.33	0.67	0.50	1	0.83
	1	1	1	0.67	0.67	0.50
	0.83	0.50	0.67	0.50	1	0
	0.83	0.50	0.83	0.33	1	0
	0.83	0.50	0.83	0.67	0.50	0.67
	1	1	0.33	1	0.83	0.50
	0.67	0.67	0.67	0.33	1	0.17
	0.67	0.83	0.83	0.17	1	1
	0.33	0.50	1	0	0.50	0.67
	0.67	0.33	0.67	0.50	1	0
	0.50	0.67	0.50	0.83	0.67	0.50
	0.67	0.50	0.33	0.67	0.33	0.50
	1	1	1	0.67	0.67	0.67
SLI	0.67	0.33	1	0.83	0.83	1
	0.67	0.67	0.33	0.83	1	0.83
	0.33	0.50	1	1	0.83	0.67
	0.67	0.67	0.50	0.67	0.67	0.67
	0.50	0.67	1	0.17	0.33	0.67
	0.50	0.67	0.50	0.50	0.33	0.50
	1	0	1	1	1	0.67
	0.50	0.50	0.83	0.17	0.67	0.67
	0.83	0.83	0.67	0.50	0.67	0.50
	1	0.83	0.17	0.67	0.33	0.67
	0.50	0.83	0.67	0.83	0.83	0.17
	1	0.50	0.83	0.50	0.17	0.83
	0.50	0.67	0.83	0.50	0.50	0.50
	1	0.50	0.33	0.67	0.33	0.83
	0.33	0.67	0.50	0.50	0.83	0.50
	0.83	0.17	0.50	0.50	0.83	0.33
	0.67	0.67	0.33	0.50	0.33	0.50
	0.83	0.50	0.50	0.67	0.67	0.50
	0.67	0.50	0.33	0.50	0.50	0.67
	0.50	0.83	0.40^*^	0.67	0.33	0.67

* One data point was missing for this participant due to program failure. Only responses to 11 test items were recorded for this participant.

Note: Correction rejections rate (i.e., ungrammatical strings judged as ungrammatical) = 1− false positive rate; Miss rate (i.e., grammatical strings judged as ungrammatical) = 1− hit rate.

**Table A2:** **Language composite scores and number of non-adjacent pairs learned (100% accuracy) for each participant in the three conditions**.

	*****X* = 2****		*****X* = 12****		*****X* = 24****	
	**Language**^a^****	**Items**^b^****	**Language**^a^****	**Items**^b^****	**Language**^a^****	**Items**^b^****
NL	0.69	0	−0.32	0	0.79	0
	−0.04	0	0.53	0	0	0
	1.75	0	−0.11	0	0.92	3
	−0.6	0	−0.63	0	0.97	1
	−0.52	0	1.24	0	−0.2	1
	−0.96	0	−0.16	0	−1.11	0
	0.23	0	−0.76	0	−1.06	1
	−0.77	0	0.08	1	−0.5	0
	−0.7	0	−0.97	0	−1.16	0
	−0.78	1	−0.19	1	0	0
	−0.42	1	0.46	0	0.21	3
	0.42	0	1.51	0	0.19	3
	0.55	0	0.36	0	−0.28	1
	−0.85	0	−0.6	0	0.82	0
	−0.19	0	−2.15	1	−0.76	0
	−0.32	0	−0.83	1	−0.38	2
	0.02	1	−0.89	2	−1.13	0
	−0.24	0	−0.64	0	−0.42	1
	0.3	0	0.52	0	−0.77	3
	−0.81	0	−0.73	1	−1.21	0
SLI	−1.21	0	−1.57	0	−1.06	0
	−1.58	0	−0.89	0	−1.1	1
	−1.93	0	−2.18	0	−1.47	0
	−1.31	0	−2.76	0	−1.73	0
	−1.48	0	−1.52	0	−1.14	0
	−1.9	0	−1.09	0	−2.43	0
	−1.44	0	−1.42	0	−1.38	0
	−1.82	0	−1.33	0	−1.66	1
	−1.35	0	−1.12	0	−2.32	0
	−1.02	1	−1.06	1	−1.98	0
	−1.18	3	−1.54	2	−1.52	1
	−1.52	0	−1.6	0	−1.21	0
	−2.61	0	−1.55	0	−1.17	0
	−2.3	0	−1.99	2	−1.91	0
	−0.89	1	−1.31	1	−1.32	0
	−1.43	0	−1.31	0	−1.23	0
	−0.92	1	−1.41	0	−1.74	0
	−1.28	1	−2.62	0	−1.99	0
	−2.08	0	−1.25	1	−1.14	0
	−1.82	0	−1.13	0	−1.27	0

a 8th grade language composite z scores.

b Number of items (max = 3) responded to with 100% accuracy.
